# Role of Final Kissing Balloon Inflation in Left Main Distal Bifurcation Single Stenting: Insights from Angiographic Microvascular Resistance

**DOI:** 10.3390/medicina61112062

**Published:** 2025-11-19

**Authors:** En Chen, Danqing Hu, Hong Zheng, Lianglong Chen, Wei Cai

**Affiliations:** 1 Department of Cardiology, Fujian Medical University Union Hospital, Fujian Cardiovascular Medicine Center, Fujian Institute of Coronary Artery Disease, Fujian Cardiovascular Research Center, Fujian Medical University Heart Center, Fuzhou 350001, China; chenenfj@126.com (E.C.);; 2 School of Health, Fujian Medical University, Fuzhou 350122, China

**Keywords:** left main coronary bifurcation lesion, single stenting, final kissing balloon inflation, simple crossover, coronary microcirculation, angiography-derived index of microcirculatory resistance

## Abstract

*Background and Objectives*: The roles of final kissing balloon inflation (FKBI) in single stenting remain controversial, with no prior studies evaluating its impact on angiographic restenosis from the perspective of coronary microcirculation. This study aimed to investigate whether FKBI reduced angiographic restenosis in patients treated with single stenting in the left main (LM)–left anterior descending (LAD) after propensity score matching (PSM) to balance baseline characteristics, including the pre-procedural angiography-derived index of microcirculatory resistance (AMR). Additionally, it aimed to demonstrate the temporal changes in AMR (pre-procedure, post-procedure, and follow-up) and their impact on angiographic restenosis. *Materials and Methods*: AMR was calculated based on coronary angiography from a single view of the LM–left circumflex (LCX), pre- and post-procedure and during follow-up. Long-term angiographic restenosis was assessed using percent diameter stenosis (DS%). *Results*: A total of 197 patients underwent the simple crossover, and 70 underwent FKBI, while 61 pairs were matched after the PSM. The long-term DS% in the LM and LAD was lower in the FKBI group after the PSM. The long-term AMR demonstrated an increase in the simple crossover group but stability in the FKBI group. The long-term AMR was lower post-FKBI regardless of the PSM. The pre-procedural AMR was a positive predictor of long-term LAD angiographic restenosis in the simple crossover group, but it did not show any correlation in the FKBI group. *Conclusions*: After PSM involving pre-procedural AMR, FKBI could reduce long-term angiographic restenosis in the LM and LAD following left main distal bifurcation single stenting and exhibited lower long-term AMR compared to the simple crossover group. The pre-procedural AMR predicted the future LAD progression in the simple crossover group, yet FKBI seemed to nullify the association.

## 1. Introduction

Since the concept of balloon juxtaposition was introduced in the 1980s, the final kissing balloon inflation (FKBI) technique has rapidly become established for maintaining the opening of the side branch (SB) ostium and correcting stent malapposition [1]. Its positive effects within the context of dual-stent strategies are widely acknowledged [1]. However, its role in the single-stent technique has recently been challenged, not only with potential benefits, but also with neutral effects or even deleterious outcomes when comparing single stenting with or without FKBI [2,3,4,5]. Moreover, for a left main coronary bifurcation lesion (LMCBL) with a larger reference diameter and a greater amount of myocardium involved, no definitive benefit of FKBI has been established in patients treated with single stenting [2,3,4,5]. The conflicting roles of FKBI would require more available interpretations and research.

Coronary microcirculatory dysfunction is widely recognized as a predictor for identifying patients at risk of poor clinical outcomes, and the index of microcirculatory resistance (IMR) remains the most commonly used tool to assess the status of the coronary microcirculation [6]. Previous studies have indicated an association between elevated IMR values and adverse cardiovascular events in both obstructive and non-obstructive coronary artery disease [7]. Moreover, the pre- or post-dilation and consequent distal embolization during the primary percutaneous coronary intervention (PCI) procedure has been linked to microvascular obstruction [6,8]. However, to date, no studies have reported on the link between the FKBI and coronary microcirculation in the single-stent technique, nor have they considered the role of microvascular dysfunction in explaining the inconsistent clinical outcomes of FKBI.

The conventional measurement method for IMR relies on thermodilution via a guidewire and the use of hyperemic agents, which complicates and increases the risks associated with the PCI procedure [9]. Recently, the angiography-derived index of microcirculatory resistance (AMR)—an index that is wire- and adenosine-free, computed based on a Murray’s law-based quantitative flow ratio from a single angiographic view—has gained increasing attention as a potential alternative to IMR [6,8,9,10,11,12]. However, there is a lack of data on the long-term evolution of AMR following single stenting in LMCBL and the correlation between the AMR and angiographic restenosis in LMCBL. Thus, we performed propensity score matching (PSM) to examine whether FKBI could reduce long-term angiographic restenosis in LMCBL patients who underwent the single-stent procedure after accounting for the differences in pre-procedural AMR, as compared to the simple crossover without an SB-opening procedure. Furthermore, our study investigates the temporal changes in AMR (pre-, post-procedure, and during long-term follow-up), as well as the impact of AMR on long-term angiographic restenosis in LMCBL patients treated with simple crossover techniques without SB opening and those treated with single stenting along with FKBI.

## 2. Materials and Methods

### 2.1. Study Population

This retrospective study included LMCBL patients who underwent single-stent procedures from the left main (LM) to the left anterior descending (LAD) and had completed coronary angiographies with a minimum of 6 months of follow-up. This study was conducted at Fujian Medical University Union Hospital from January 2013 to December 2021. Patients who underwent a simple crossover without an SB intervention procedure other than wire protection, as well as those who received single stenting combined with FKBI, were included in our study, regardless of whether they had undergone the proximal optimization technique (POT) or repeat POT. Meanwhile, the accessible pre-procedural, post-procedural, and long-term angiograms for AMR analysis were necessary.

The exclusion criteria were primarily as follows: (1) SB balloon dilation without subsequent final kissing; (2) a history of coronary artery bypass graft surgery; (3) total occlusion of the LM, proximal LAD or left circumflex (LCX); (4) poor-quality angiograms for AMR analyses; and (5) significant overlap in the segment of LMCBL for reliable lesion measurements. The clinical characteristics, laboratory tests, and details of the intervention procedures were also collected from the included LMCBL patients. Furthermore, this research was conducted in compliance with the Declaration of Helsinki and the Ethical Review Measures for Life Sciences and Medical Research Involving Human Subjects. Additionally, this study was reviewed and approved by the Ethics Committee of Fujian Medical University Union Hospital (No. 2025KY120), and informed consent was waived due to its retrospective nature.

### 2.2. AMR and Lesion Measurements

A single-view AMR analysis was conducted as previously described [8,9,13], using AngioPlus Core software (version V3; Shanghai Pulse Medical Technology, Shanghai, China) by 2 experienced and certified analysts blinded to interventional strategies and clinical data. The AMR measurements were performed in the optimal angiogram of LM-LCX pre- and post-procedure and during follow-up.

The long-term angiographic restenosis was assessed based on the percent diameter stenosis (DS%) in three bifurcation segments of the LMCBL during long-term follow-up, also using AngioPlus Core software.

### 2.3. Statistical Analysis

Statistical analysis was conducted using the SPSS software (version 27; SPSS Inc, Chicago, IL, USA). The Shapiro–Wilk normality test was applied, and continuous variables were presented as mean ± standard deviation and median (interquartile range). For comparisons of continuous variables between two groups with normality and homogeneity of variance, the independent samples *t*-test was performed. Temporal changes in AMR (pre-procedure, post-procedure, and during follow-up) were examined using the matched Friedman test. Differences in AMR stratified by SB interventional strategies (simple crossover or FKBI) were determined using the independent Mann–Whitney U test. Categorical variables were expressed as absolute frequency and percentage, with differences compared using the Pearson chi-square test.

To mitigate the impact of baseline disparities in clinical, bifurcation morphology, and coronary microcirculation characteristics on long-term angiographic restenosis, a 1:1 PSM was employed to pair patients from the simple crossover and FKBI groups. The matching criteria included age; sex; clinical presentation (unstable angina or acute myocardial infarction); diabetes mellitus; hypertension; dyslipidemia; smoking status; body mass index (BMI); low-density lipoprotein cholesterol (LDL-C); high-density lipoprotein cholesterol (HDL-C); cholesterol; serum creatinine; fasting blood glucose; left ventricular ejection fraction (LVEF); duration of follow-up; proportion of intermediate coronary artery; the use of intravascular ultrasound/optical coherence tomography (IVUS/OCT); pre-procedural DS% in the LM, LAD, and LCX; and pre-procedural AMR. The ratio value was set to 1, with a caliper value of 0.02.

The linear regression analysis was conducted to explore whether the AMR significantly contributed to the prediction of long-term DS%. Statistical significance was defined as a two-sided *p* value of less than 0.05.

## 3. Results

### 3.1. Baseline Characteristics

A total of 267 patients treated with the single-stent technique were enrolled in this study, stratified according to the conjunctive SB interventional strategies, with 197 patients in the simple crossover group and 70 patients in the FKBI group. Additionally, 61 pairs of patients were matched by PSM. All included patients were treated with second-generation drug-eluting stents, with 219 (82%) being male and a mean age of 65.3 years. The follow-up time was 382 (361–459) days. As indicated in Table 1, before the PSM, hypertension and the utilization rates of intracoronary imaging devices (IVUS/OCT) were more prominent in the FKBI group. However, after PSM, there were no significant differences in the baseline characteristics between the two groups.

### 3.2. Lesion Data

As presented in Table 2, compared to the simple crossover group before PSM, the DS% in the LAD before the procedure was lower in the FKBI group (simple crossover 63.8 (54.9–68.8) vs. FKBI 58.4 (47–66.4), *p* = 0.012) but with a higher DS% in the LCX before the procedure (simple crossover 19.4 (13.2–26.8) vs. FKBI 26.3 (15.6–38), *p* = 0.002). After the PSM, the pre-procedural lesion stenosis was equivalent between the two groups.

**Figure 1:**
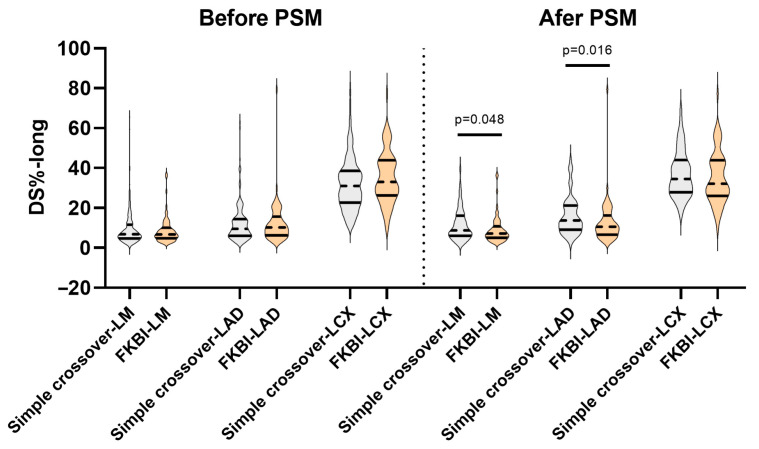
Discrepancies in long-term DS% between the simple crossover and FKBI groups. DS%: percent diameter stenosis; FKBI: final kissing balloon inflation; PSM: propensity score matching; DS%-long: long-term DS%; LM: left main; LAD: left anterior descending; and LCX: left circumflex.

As exhibited in Figure 1, the long-term DS% (DS%-long) in three bifurcation segments of the LMCBL was comparable between the two groups before the PSM. However, after the PSM, the DS%-long in the LM and LAD were lower in the FKBI group (LM: simple crossover 8.8 (6–16) vs. FKBI 7.2 (5–10.8), *p* = 0.048; LAD: simple crossover 13.7 (9.1–21.2) vs. FKBI 10.6 (6.6–16.2), *p* = 0.016). It is worth mentioning that, immediately post-procedure, the DS% in the LCX was lower in the FKBI group after the PSM (simple crossover 29.5 (24.5–36.1) vs. FKBI 25.6 (19.2–34.8), *p* = 0.043). However, during the long-term follow-up, no significant benefit in angiographic restenosis in the LCX was detected in the FKBI group (simple crossover 34.5 (27.9–44) vs. FKBI 32.1 (26–43.9), *p* = 0.293).

### 3.3. AMR Analyses

**Figure 2:**
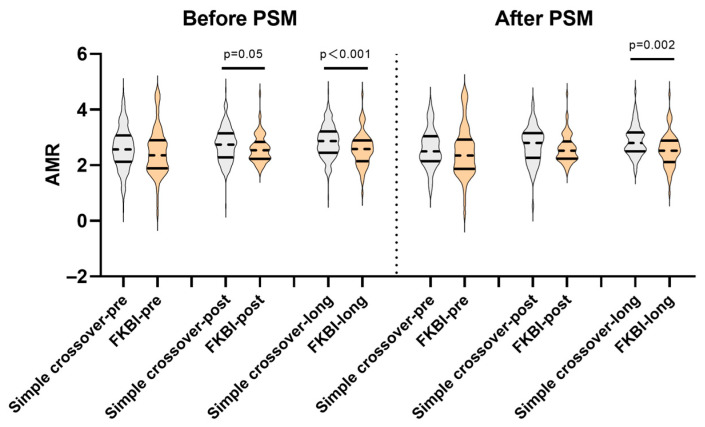
Comparisons of pre-procedural, post-procedural, and long-term AMR between the simple crossover and FKBI groups. AMR: angiography-derived index of microcirculatory resistance; FKBI: final kissing balloon inflation; PSM: propensity score matching; Simple crossover-pre, -post, and -long: AMR before, post-procedure, and during long-term follow-up in the simple crossover group; FKBI-pre, -post, and -long: AMR before, post-procedure, and during long-term follow-up in the FKBI group.

The details of the AMR between the two groups are presented in Table 2 and Table 3. As depicted in Figure 2, there were no significant differences in the pre-procedural AMR between the two groups, whether before or after the PSM. But when combined with the FKBI procedure, the post-procedural and long-term AMR appeared to be lower before the PSM (post-procedural AMR: simple crossover 2.74 (2.28–3.15) vs. FKBI 2.54 (2.23–2.84), *p* = 0.05; long-term AMR: simple crossover 2.87 (2.45–3.22) vs. FKBI 2.59 (2.15–2.89), *p* < 0.001). Even after performing the PSM, the long-term AMR was also lower in the FKBI group (simple crossover 2.8 (2.5–3.18) vs. FKBI 2.52 (2.12–2.89), *p* = 0.002).

**Figure 3:**
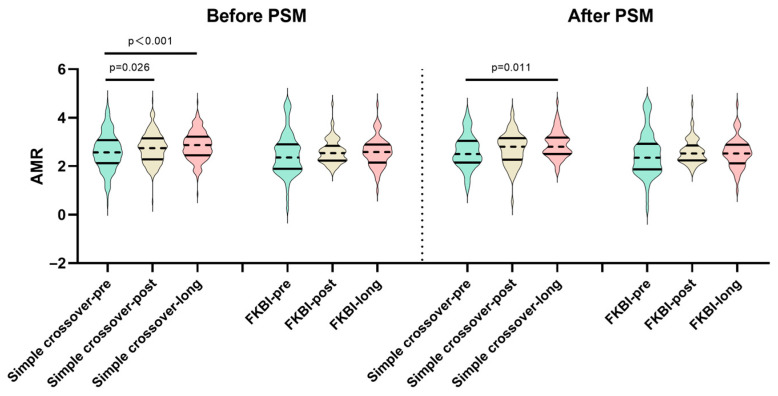
Temporal changes in AMR among LMCBL patients, stratified by the simple crossover and FKBI groups. AMR: angiography-derived index of microcirculatory resistance; LMCBL: left main coronary bifurcation lesion; FKBI: final kissing balloon inflation; PSM: propensity score matching; Simple crossover-pre, -post, and -long: AMR before, post-procedure, and during long-term follow-up in the simple crossover group; FKBI-pre, -post and -long: AMR before, post-procedure, and during long-term follow-up in the FKBI group.

The temporal changes in the AMR are shown in Table 3 and Figure 3. In the FKBI group, whether before or after the PSM, no differences were observed among the pre-procedural, post-procedural, and long-term AMR. However, the simple crossover group exhibited a higher post-procedural and long-term AMR, when compared to the pre-procedural AMR before the PSM (before procedure: 2.57 (2.13–3.07) vs. post-procedure 2.74 (2.28–3.15), *p* = 0.026; before procedure: 2.57 (2.13–3.07) vs. long-term follow-up: 2.87 (2.45–3.22), *p* < 0.001). Furthermore, after the PSM, the simple crossover group also indicated a higher long-term AMR (before procedure: 2.5 (2.15–3.04) vs. long-term follow-up: 2.8 (2.5–3.18), *p* < 0.011).

### 3.4. Impact of AMR on Long-Term Angiographic Restenosis

**Figure 4:**
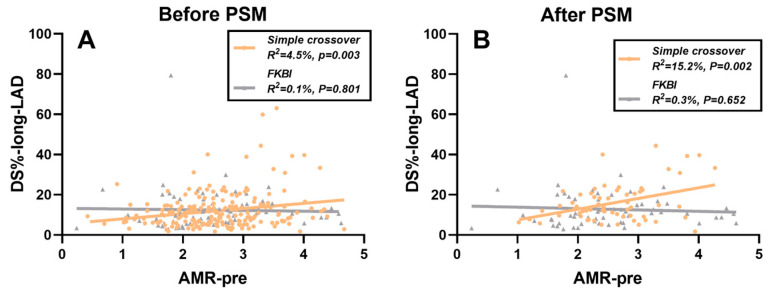
Correlations of long-term DS% in LAD and pre-procedural AMR. (**A**) Correlation of long-term DS% in LAD and pre-procedural AMR before PSM, stratified by the simple crossover and FKBI groups. (**B**) Correlation of long-term DS% in LAD and pre-procedural AMR before PSM, stratified by the simple crossover and FKBI groups after PSM. DS%: percent diameter stenosis; LAD: left anterior descending; AMR: angiography-derived index of microcirculatory resistance; PSM: propensity score matching; FKBI: final kissing balloon inflation; DS%-long-LAD: long-term DS% in LAD; and AMR-pre: pre-procedural AMR.

After evaluating the benefits of FKBI by accounting for the pre-procedural AMR and examining the temporal changes in AMR, a linear regression analysis was conducted to investigate the effect of AMR on long-term angiographic restenosis. As shown in Table 4, the pre-procedural AMR seemed to significantly influence the long-term DS% in the LAD within the simple crossover group, irrespective of whether PSM was performed (before PSM: R^2^ = 4.5%, *p* = 0.003, Figure 4A; after PSM: R^2^ = 15.2%, *p* = 0.002, Figure 4B). However, in the FKBI group, no significant linear correlation was detected. Additionally, no correlation was identified between the AMR and the long-term DS% in the LM and LCX in either group, before or after PSM, as indicated in Supplementary Table S1.

## 4. Discussion

To the best pf our knowledge, this is the first study to introduce coronary microcirculation into the context of coronary bifurcation lesions, interpreting the controversial roles of FKBI in LMCBL patients treated with the single-stent technique from the perspective of AMR. The main findings in our study were as follows: (1) The long-term DS% in the LM and LAD were lower in the FKBI group after the PSM involving pre-procedural AMR. (2) The long-term evolution of the AMR differed between the two groups, with an increase in AMR during the follow-up in the simple crossover group but stability in the FKBI group. (3) Compared to the simple crossover group, the long-term AMR was lower following the FKBI procedure both before and after the PSM. (4) The pre-procedural AMR was a positive predictor of long-term LAD angiographic restenosis in the simple crossover group both before and after the PSM, but it did not show any correlation in the FKBI group.

For years, the FKBI has been considered an optimization technique to correct proximal main vessel (MV) stent malapposition and limit strut obstructions in the SB ostium in single stenting [1]. Following the FKBI, the minimal lumen diameters (MLDs) in the MV and SB would also be larger compared to those who did not perform the FKBI, which was associated with less target lesion revascularization (TLR) in the COBIS II study [14]. However, this contradicted the COBIS I study, which showed an increase in the cross-sectional area from 5.2 ± 1.2 mm^2^ to 5.6 ± 1.2 mm^2^ after FKBI, yet it resulted in more TLR [15]. Furthering the COBIS III study in the update, additional SB-opening procedures, including FKBI, demonstrated neutral effects compared to the simple crossover alone in patients treated with the single-stent technique [16]. Focusing on the LMCBL patients treated with the single-stent technique in conjunction with FKBI in the ASAN-MAIN registry [3] and the sub-analysis of the EXCEL Trial [5], no clinical benefits were detected.

Except for the extensive use of IVUS, which masked the benefit of FKBI in a sub-analysis of the EXCEL Trial [5], and patients with a preference for angina over myocardial infarction in previous randomized controlled trials as reported by He et al. [17], as well as the inappropriate application of FKBI noted in the PROPOT trial [18], the main reason for the unsatisfactory results of FKBI was attributed to the overdistension of the proximal MV into an abnormal elliptical shape, increasing the risks of proximal marginal dissection and future restenosis [18,19]. In the study reported by Bianchini et al. [19], even when combined with final proximal MV optimization after simultaneous balloon inflation, the stent ellipticity in the proximal MV was still prominent in adult healthy swine. However, the higher proximal MV ellipticity was associated with improved stent apposition and SB ostium scaffolding. The conflicting roles of FKBI would require more available interpretations.

In our study, we found that the observed differences in clinical outcomes among previous studies may be explained by the inclusion of patients with varying statuses of pre-procedural coronary microcirculation. Before the PSM involving the pre-procedural AMR, no benefit in long-term angiographic restenosis had been detected in the FKBI group. However, after the PSM, the long-term DS% in the LM and LAD was lower in patients who underwent the FKBI procedure. This suggested that if the enrolled patients in previous studies were matched based on their pre-procedural status of coronary microcirculation, it could enable a clearer examination of the effectiveness of FKBI when using the single-stent technique.

Actually, many cardio-interventional specialists have been aware of the significance of assessing microcirculation in patients with coronary artery disease [20]. Initially proposed by Fearon et al. [21], IMR, the novel index for evaluating coronary microcirculation function, has been confirmed to possess a clinical application value in cases of acute ST elevation myocardial infarction (STEMI), angina, and cardiomyopathy [22,23,24]. However, traditional IMR methods require the administration of adenosine to sustain coronary hyperemia, which is time-consuming and labor-intensive, limiting their use in LMCBL patients [25]. With advancements in angiography-based microcirculation evaluation methods, the AMR algorithm has come into the researchers’ focus. It is based on the Murray bifurcation fractal law and calculates and simulates maximal hyperemia from a single angiographic image, thereby facilitating a rapid coronary microcirculation assessment during angiography [26].

In recent years, AMR has demonstrated a significant potential for broader clinical application. After confirming the strong correlation with invasive IMR, by Fan et al. [11], AMR has been widely used in STEMI patients who undergo primary PCI to evaluate drug-coated balloons [6] or post-dilatation [8], to predict composite adverse clinical events [27] or new-onset heart failure [9], as well as to reveal the association between the triglyceride–glucose index and AMR [28]. Moreover, AMR was utilized not only in patients with STEMI but also in those with unstable anginas to assess the predictive value of metabolic risk factors for developing microcirculation disorders [10] and in patients experiencing ischemic symptoms without obstructive coronary artery disease to confirm the AMR’s diagnostic precision [29]. Even in patients undergoing transcatheter aortic valve replacement, AMR had also confirmed its prognostic value with adverse clinical outcomes [13]. However, it was regrettable that neither traditional IMR nor AMR had been previously applied to assess the coronary microcirculation in the context of coronary bifurcation lesions.

Indeed, micro-embolization caused by stenting or balloon dilation in interventional procedures was associated with coronary microvascular dysfunction, as indicated by increased IMR or AMR values [6]. However, the long-term evolution of the coronary microcirculation remained unclear. Our study directly addressed this gap by demonstrating a divergent long-term evolution of AMR between the two procedural strategies: while the simple crossover group exhibited a significant increase in AMR during the follow-up, indicative of progressive microvascular impairment, the FKBI group maintained stable AMR values. This critical finding, consistent both before and after the PSM, suggested that FKBI conferred a protective effect on the microcirculation. Consequently, our results position AMR not merely as an outcome measure but as a valuable functional endpoint for evaluating procedural optimization. The stabilization of AMR by FKBI provided a crucial physiological correlate to its anatomical benefit of reducing restenosis, thereby offering a more comprehensive paradigm for assessing procedural success beyond conventional lumenography.

Upon examining the impact of AMR on long-term angiographic restenosis, it was interesting that the pre-procedural AMR served as a positive predictor for future LAD progression in patients treated with the simple crossover alone both before and after PSM, yet it did not in the FKBI group. This indicated that combining with FKBI would offset the adverse effects of a large pre-procedural AMR. Except for the potential stabilization of AMR by FKBI, the explanations for the offset effect might partly lie in the increased MLD and better apposition in the MV by FKBI. However, these specific anatomical mechanical benefits remained speculative within the context of our study, due to the lack of systematic intracoronary imaging (IVUS/OCT) data. The observed benefits of FKBI should be regarded as a demonstrated positive functional effect on the microcirculation. We postulated that this was a downstream consequence of the superior mechanical optimization achieved by FKBI, such as better stent expansion and apposition. A definitive linkage among the technique, anatomical perfection, and functional microcirculatory benefits requires validation in future prospective, imaging-guided studies.

Our study also interestingly indicated that the immediate post-procedural benefit in reducing the DS% in the LCX achieved by FKBI did not translate into a significant reduction in long-term angiographic restenosis. This apparent dissociation between the acute post-procedural gain and the long-term outcome merited consideration. The procedure of FKBI might induce a localized vascular injury and an inflammatory response at the delicate ostium, potentially stimulating a healing process that counteracted the early benefit. Additionally, with the unique hemodynamic environment in the ostium of the LCX, turbulent flow and altered shear stress would promote progressive neointimal hyperplasia. Hence, compared to the modest and potentially unstable local effect at the LCX ostium, the value of FKBI in microcirculatory improvement in a provisional stenting strategy for LMCBL might lie in better anatomical mechanical optimization in the critical MV to sustain the vast territory perfusion into the major epicardial vessel, as reflected by the stable AMR in the FKBI group.

This study had several limitations that should be acknowledged. First, its retrospective, single-center design and the limited sample size inherently constrained the statistical power and limited the generalizability of our findings. This study only encompassed individuals with accessible follow-up angiograms. Even though the subjects were selected in a consecutive manner, a selection bias within the included population was inevitable. Furthermore, a potential selection bias was inevitable due to the exclusion of patients who ultimately required a dual-stent technique for limiting the SB flow. Consequently, our conclusions regarding the role of FKBI were strictly applicable to a selected population with low-to-moderate-complexity LMCBL that were amenable to a provisional single stenting strategy and should not be extrapolated to more complex anatomies. Second, our study lacked systematically accurate data on the performance of POT in the retrospective design. Thus, we could not rule out that the uneven use of the POT between the groups might have influenced the results. Third, despite the PSM, the residual confounding from unmeasured variables represents a fundamental limitation, as factors like the bifurcation angle, detailed plaque burden, and nuanced procedural techniques could not be balanced. Fourth, the primary endpoint in our study was the angiographic evaluation not the clinical outcome, which precluded a definitive conclusion on clinical superiority. Fifth, due to the infrequent use of intracoronary imaging devices, data from intracoronary imaging were not available, which constituted a critical limitation for a mechanistic interpretation of our results. Finally, the assessment of the AMR was derived from single-view coronary angiography. While practical, this method might not be fully equivalent to pressure wire-based indices. Therefore, our results should be interpreted as generating hypotheses within the context of a high-volume tertiary center and require validation in larger, multicenter, and prospective cohorts.

## 5. Conclusions

After PSM involving the pre-procedural AMR, FKBI was associated with a favorable reduction in long-term angiographic restenosis in the LM and LAD following left main distal bifurcation single stenting and demonstrated a lower long-term AMR compared to the simple crossover group. The pre-procedural AMR was a positive predictor of future LAD progression in the simple crossover group, which could be mitigated by FKBI. The current preliminary data could serve as a foundation for future research into the use of AMR during FKBI procedural planning.

## Figures and Tables

**Table 1:** Baseline characteristics of the included patients.

	Before PSM			After PSM		
	Simple Crossover (n = 197)	FKBI (n = 70)	*p* Value	Simple Crossover (n = 61)	FKBI (n = 61)	*p* Value
Age, years	65 ± 10.3	66.3 ± 8.8	0.342	68.1 ± 9.6	66 ± 9.1	0.231
Male gender, n (%)	160 (81.2%)	59 (84.3%)	0.566	50 (82%)	50 (82%)	1
BMI, kg/m^2^	24.2 (22.1–26.1)	24.2 (22.4–26.3)	0.941	24.2 (22.7–26.3)	24.2 (22.7–26.4)	0.955
Current UA, n (%)	134 (68%)	54 (77.1%)	0.151	60 (82%)	46 (75.4%)	0.377
Current MI, n (%)	63 (32%)	16 (22.9%)	0.151	11 (18%)	15 (24.6%)	0.377
Prior MI, n (%)	27 (13.7%)	12 (17.1%)	0.484	8 (13.1%)	8 (13.1%)	1
Diabetes mellitus, n (%)	70 (35.5%)	31 (44.3%)	0.195	25 (41%)	26 (42.6%)	0.854
Hypertension, n (%)	123 (62.4%)	54 (77.1%)	0.025	47 (77%)	45 (73.8%)	0.674
Dyslipidemia, n (%)	75 (38.1%)	25 (35.7%)	0.726	25 (41%)	23 (37.7%)	0.711
Current smoker, n (%)	70 (35.5%)	26 (37.1%)	0.809	26 (42.6%)	21 (34.4%)	0.352
LDL-C, mmol/L	2.6 (2–3.4)	2.5 (1.9–3.3)	0.737	2.8 (2.1–3.5)	2.5 (1.9–3.6)	0.472
HDL-C, mmol/L	1 (0.8–1.2)	1 (0.8–1.1)	0.491	1 (0.9–1.1)	1 (0.8–1.2)	0.856
Cholesterol, mmol/L	4 (3.3–4.9)	3.8 (3.3–4.8)	0.953	4 (3.4–5)	3.9 (3.3–5.2)	0.519
Serum creatinine, mmol/L	80 (70–93)	81 (69.8–95)	0.625	80 (70.5–91)	83 (70.5–95)	0.527
Fasting blood glucose, mmol/L	5.6 (4.9–7)	5.9 (5.2–7.1)	0.21	5.7 (4.9–6.7)	5.9 (5.3–7.4)	0.114
LVEF, %	62.5 (54.9–68.4)	62.4 (56.6–69.2)	0.58	62.9 (58.4–69)	62.3 (56.3–69)	0.822
Follow-up, days	382 (361–458.5)	378 (353.8–483.5)	0.693	393 (363–592.5)	378 (353–456.5)	0.231
Intermediate coronary artery, n (%)	32 (16.2%)	12 (17.1%)	0.862	9 (14.8%)	10 (16.4%)	0.803
IVUS/OCT, n (%)	52 (26.4%)	35 (50%)	<0.001	20 (32.8%)	28 (45.9%)	0.138

Values are n (%), mean ± standard deviation, and median (interquartile range). PSM: propensity score matching; FKBI: final kissing balloon inflation; BMI: body mass index; UA: unstable angina; MI: myocardial infarction; LDL-C: low-density lipoprotein cholesterol; HDL-C: high-density lipoprotein cholesterol; LVEF: left ventricular ejection fraction; and IVUS/OCT: intravascular ultrasound/optical coherence tomography.

**Table 2:** Angiographic characteristics of the included patients.

	Before PSM			After PSM		
	Simple Crossover (n = 197)	FKBI (n = 70)	*p* Value	Simple Crossover (n = 61)	FKBI (n = 61)	*p* Value
AMR-pre	2.57 (2.13–3.07)	2.36 (1.89–2.9)	0.178	2.5 (2.15–3.04)	2.35 (1.87–2.93)	0.275
AMR-post	2.74 (2.28–3.15)	2.54 (2.23–2.84)	0.05	2.8 (2.27–3.16)	2.52 (2.24–2.86)	0.194
AMR-long	2.87 (2.45–3.22)	2.59 (2.15–2.89)	<0.001	2.8 (2.5–3.18)	2.52 (2.12–2.89)	0.002
DS%-pre in LM	45.7 (25.3–61.2)	51.3 (29.3–64.2)	0.165	51.7 (28.9–61.2)	50.8 (27.8–64.8)	0.862
DS%-pre in LAD	63.8 (54.9–68.8)	58.4 (47–66.4)	0.012	58.8 (51.4–67.4)	58.6 (48.4–68.1)	0.737
DS%-pre in LCX	19.4 (13.2–26.8)	26.3 (15.6–38)	0.002	25 (17.2–35.7)	25.8 (15.4–31.1)	0.63
DS%-post in LM	3.2 (0–5.7)	2.5 (0–5)	0.313	4.2 (0–9)	2.4 (0–5.1)	0.057
DS%-post in LAD	5.7 (3.2–8.5)	6.4 (3.2–10.1)	0.258	7.6 (5.6–11.7)	6.4 (3.3–10.2)	0.079
DS%-post in LCX	25.9 (19.8–33.8)	25.7 (19.2–37.1)	0.973	29.5 (24.5–36.1)	25.6 (19.2–34.8)	0.043
DS%-long in LM	6.8 (4.6–11.6)	6.8 (4.8–10.1)	0.987	8.8 (6–16)	7.2 (5–10.8)	0.048
DS%-long in LAD	9.6 (6–14.4)	10.2 (6.2–15.6)	0.656	13.7 (9.1–21.2)	10.6 (6.6–16.2)	0.016
DS%-long in LCX	31 (22.6–38.6)	33.1 (26.2–43.9)	0.083	34.5 (27.9–44)	32.1 (26–43.9)	0.293

Values are median (interquartile range). PSM: propensity score matching; FKBI: final kissing balloon inflation; AMR: angiography-derived index of microcirculatory resistance; AMR-pre, AMR-post, and AMR-long: AMR before, immediately after procedure, and during long-term follow-up; DS%: percent diameter stenosis; DS%-pre, DS%-post, and DS%-long: DS% before, immediately after procedure, and during long-term follow-up; LM: left main; LAD: left anterior descending; and LCX: left circumflex.

**Table 3:** Temporal changes in AMR.

		Pre-Procedure	Post-Procedure	Follow-Up	*p* Value
Before PSM	Simple crossover	2.57 (2.13–3.07)	2.74 (2.28–3.15)	2.87 (2.45–3.22)	<0.001
	FKBI	2.36 (1.89–2.9)	2.54 (2.23–2.84)	2.59 (2.15–2.89)	0.68
After PSM	Simple crossover	2.5 (2.15–3.04)	2.8 (2.27–3.16)	2.8 (2.5–3.18)	0.011
	FKBI	2.35 (1.87–2.93)	2.52 (2.24–2.86)	2.52 (2.12–2.89)	0.664

Values are median (interquartile range). PSM: propensity score matching; FKBI: final kissing balloon inflation; and AMR: angiography-derived index of microcirculatory resistance.

**Table 4:** Linear regression assessing the impact of AMR on the predictions of long-term DS% in LAD.

	Variables Predicting DS%-Long in LAD	B	SE	β	t	*p* Value	VIF	R	R^2^	D-W
Before PSM	Simple crossover							0.227	0.052	2.036
								F = 5.278	*p* = 0.006	
	AMR-pre	2.893	0.891	0.239	3.247	0.001	1.107			
	AMR-post	−1.258	1.111	−0.083	−1.132	0.259	1.107			
	FKBI							0.046	0.002	1.401
								F = 0.072	*p* = 0.931	
	AMR-pre	−0.321	1.43	−0.028	−0.225	0.823	1.008			
	AMR-post	−0.702	2.483	−0.035	−0.283	0.778	1.008			
After PSM	Simple crossover							0.398	0.158	1.887
								F = 5.45	*p* = 0.007	
	AMR-pre	4.921	1.75	0.36	2.811	0.007	1.13			
	AMR-post	1.24	1.834	0.087	0.676	0.502	1.13			
	FKBI							0.075	0.006	2.03
								F = 0.162	*p* = 0.85	
	AMR-pre	−0.645	1.525	−0.056	−0.423	0.674	1.005			
	AMR-post	−0.959	2.735	−0.046	−0.351	0.727	1.005			

AMR: angiography-derived microcirculatory resistance; DS%: percent diameter stenosis; LAD: left anterior descending; DS%-long: long-term DS%; PSM: propensity score matching; AMR-pre and AMR-post: AMR before and immediately after procedure; and FKBI: final kissing balloon inflation.

## Data Availability

The data will be made available upon reasonable request.

## Supplementary Materials

The following supporting information can be downloaded at https://www.mdpi.com/article/10.3390/medicina61112062/s1, Table S1: Linear regression assessing the impact of AMR on the predictions of long-term DS% in LM and LCX.

## Abbreviations

The following abbreviations are used in this manuscript:

FKBI	final kissing balloon inflation
SB	side branch
LMCBL	left main coronary bifurcation lesion
IMR	index of microcirculatory resistance
PCI	percutaneous coronary intervention
AMR	angiography-derived index of microcirculatory resistance
PSM	propensity score matching
LM	left main
LAD	left anterior descending
POT	proximal optimization technique
LCX	left circumflex
DS%	percent diameter stenosis
BMI	body mass index
LDL-C	low-density lipoprotein cholesterol
HDL-C	high-density lipoprotein cholesterol
LVEF	left ventricular ejection fraction
IVUS/OCT	intravascular ultrasound/optical coherence tomography
DS%-long	long-term DS%
MV	main vessel
MLD	minimal lumen diameters
TLR	target lesion revascularization
STEMI	ST elevation myocardial infarction
