# Supplementary material for: Role of Final Kissing Balloon Inflation in Left Main Distal Bifurcation Single Stenting: Insights from Angiographic Microvascular Resistance
Source: Medicina (Kaunas). 2025 Nov 19;61(11):2062. doi: 10.3390/medicina61112062 (PMC12654271; doi:10.3390/medicina61112062)
Supplement: Supplementary file 1 [file medicina-61-02062-s001.zip › medicina-3937047-supplementary.pdf]

Supplementary Table S1 Linear regression assessing the impact of AMR on the predictions of long-term DS% in LM and LCX.

	Variables predicting DS%-long in LM	B	SE	β	t	P value	VIF	R	R2	D-W
Before PSM	Simple crossover							0.039<br>F=0.15	0.002<br>p=0.861	1.954
	AMR-pre	-0.265	0.841	-0.024	-0.315	0.753	1.107			
	AMR-post	-0.343	1.050	-0.025	-0.327	0.744	1.107			
	FKBI							0.058<br>F=0.114	0.003<br>p=0.892	2.232
	AMR-pre	0.328	0.93	0.043	0.353	0.725	1.008			
	AMR-post	0.468	1.615	0.035	0.29	0.773	1.008			
After PSM	Simple crossover							0.077<br>F=0.171	0.006<br>p=0.843	1.97
	AMR-pre	0.65	1.549	0.058	0.42	0.676	1.13			
	AMR-post	0.391	1.622	0.034	0.241	0.81	1.13			
	FKBI							0.034<br>F=0.033	0.001<br>p=0.968	2.149
	AMR-pre	0.242	0.995	0.032	0.244	0.808	1.005			
	AMR-post	0.108	1.785	0.008	0.06	0.952	1.005			

	Variables predicting DS%-long in LCX	B	SE	β	t	P value	VIF	R	R2	D-W
Before PSM	Simple crossover							0.154<br>F=2.36	0.024<br>p=0.097	1.73
	AMR-pre	-0.656	1.291	-0.038	-.509	0.612	1.107			
	AMR-post	-2.976	1.610	-0.138	-1.848	0.066	1.107			
	FKBI							0.13<br>F=0.572	0.017<br>p=0.567	2.11
	AMR-pre	1.609	1.839	0.106	0.875	0.385	1.008			
	AMR-post	1.702	3.193	0.065	0.533	0.596	1.008			
After PSM	Simple crossover							0.242<br>F=1.799	0.058<br>p=0.175	1.855
	AMR-pre	-2.258	2.275	-0.134	-0.993	0.325	1.13			
	AMR-post	-2.823	2.383	-0.16	-1.184	0.241	1.13			
	FKBI							0.104<br>F=0.316	0.011<br>p=0.73	2.332
	AMR-pre	0.965	1.896	0.067	0.509	0.613	1.005			
	AMR-post	1.946	3.402	0.075	0.572	0.57	1.005			

AMR: angiography-derived microcirculatory resistance; DS%: percent diameter stenosis; LM: left main; LCX: left circumflex; DS%-long: long-term DS%; PSM: propensity score matching; AMR-pre and AMR-post: AMR before and immediately after procedure; FKBI: final kissing balloon inflation.
